# Arch expansion with the Invisalign system: Efficacy and predictability

**DOI:** 10.1371/journal.pone.0242979

**Published:** 2020-12-10

**Authors:** Ignacio Morales-Burruezo, José-Luis Gandía-Franco, Juan Cobo, Arturo Vela-Hernández, Carlos Bellot-Arcís

**Affiliations:** 1 Orthodontics Teaching Unit, Department of Stomatology, Faculty of Medicine and Dentistry, University of Valencia, Valencia, Spain; 2 Department of Stomatology, Faculty of Medicine and Dentistry, University of Valencia, Valencia, Spain; 3 Department of Surgery and Medical-Surgical Specialties, Area of Orthodontics, Medical and Dental School, Instituto Asturiano de Odontologia, University of Oviedo, Oviedo, Spain; Danube Private University, AUSTRIA

## Abstract

**Background:**

In adult patients, treatment of skeletal crossbite requires combined treatment by fixed or removable appliances and orthognathic surgery. In cases of dentoalveolar crossbite, expansion can be achieved with fixed multibrackets and removable transparent aligners. Various researchers have already assessed the Invisalign system’s predictability for arch expansion. However, most of this research was conducted using older appliances, making it necessary to assess the characteristics of the updated system SmartTrack.

**Material and methods:**

A sample of 114 patients with transverse malocclusion were treated with SmartTrack. The predictability of the system’s software (Clincheck) was assessed by comparing planned measurements (width of canines, premolars and molars rotations and inclinations) with the real measurements achieved at the end of the first treatment phase. Measurements were imported to Clincheck software to create three data sets; T1: initial measurements at start of treatment; T2: Clincheck predicted measurements at end of first treatment phase; T3: measurements taken at start of the second treatment phase.

**Results:**

Widths underwent significant advances as a result of treatment. For all widths, virtual planning obtained prognoses of greater expansion than actually achieved: a mean of 0.63 mm more expansion at the canine level (p<0.001), 0.77 mm at first premolar (p<0.001), 0.81 at second premolar (p<0.001), 0.69 mm at first molar (p<0.001), and 0.25 mm at second molar (p = 0.183). All the treatment plan’s estimations, with the exception of the second molar, were significantly higher than the actual outcomes.

**Conclusions:**

Aligners are an effective tool for producing arch expansion, being more effective in premolar area and less effective in canine and second molar area. Predictability was reasonable for expansion movement. Overcorrection should be considered at the virtual planning stage in order to obtain the expected outcomes.

## Introduction

In the transverse plane, occlusion is considered correct when the palatal cusps in the upper posterior regions occlude into the fossae of the lower posterior teeth. When the vestibular cusps in upper posterior regions occlude into the fossae of the lower posterior teeth, this produces the malocclusion known as posterior crossbite. This type of malocclusion may be of skeletal origin, whereby the dentoalveolar processes are correctly positioned in relation to the bone base but the base presents either maxillary skeletal hypoplasia or mandibular skeletal hyperplasia (or both). When the malocclusion is of dental origin, the bone base will present a correct transversal proportion but irregular dentoalveolar processes. Different articles of epidemiological research place the prevalence of crossbite between 1% and 21%, these variations may depend on several issues (country, social class, age of the subjects…) [1, 2].

Regarding the malocclusion in general, it is thought that the need for orthodontic treatment among teenagers aged from 12–15 years is around 20% [3], among adults aged 35–44 years it is 31.3% according to the Dental Aesthetic Index (DAI), but 19.2% according to the Index of Orthodontic Treatment Need (IOTN), and 21% according to patients’ subjective perception [4].

Treatment of dental crossbite consists of dentoalveolar expansion. When children and teenagers require this treatment, it routinely involves the use of a removable Hawley plate with expansion screw or fixed Quad-Helix appliance, while in cases of skeletal origin a Hyrax palatal expander is bonded to metal or acrylic resin bands. This produces a rupture of the mid-palatal suture achieving expansion of the bone bases. In adult patients, treatment of crossbite of skeletal origin requires combined treatment by fixed (multibrackets) or removable (aligners) appliances and orthognathic surgery [5]. In cases of dentoalveolar crossbite, expansion can be achieved with fixed multibrackets and removable transparent aligners.

Various researchers have already assessed the Invisalign system’s transparent aligners (Align Technology, Santa Clara, CA, USA) and the predictability of its treatment planning software (Align Technology, Santa Clara, CA, USA) for arch expansion [6–13]. While some authors have evaluated the efficacy of tooth movement with clear aligners [14], others have compared them with fixed appliance therapies [15, 16]. However, most of this research was conducted using the older system EX30, which has since been replaced by SmartTrack (Align Technology, Santa Clara, CA, USA), making it necessary to assess the characteristics of the updated system. At the same time, most of the earlier research lacked scientific quality and rigor due to methodological bias, small samples, lack of method error analysis, or deficient statistical analysis, moderate or high risk of bias, and high heterogeneity [17, 18].

Therefore, the objectives of this study were, firstly, to determine the efficacy of the Invisalign system for arch expansion, and secondly to assess the predictability of the measurements planned by Clincheck software for the use of the transparent aligners at the end of the first treatment phase. The null hypothesis was that aligners are not an effective tool for producing arch expansion.

## Material and methods

The study protocol was approved by the ethics committee of the University of Valencia (procedural registration #1269428). All patients were informed of the study design beforehand and informed consent was given prior to participating.

This retrospective study investigated patients, who were treated by two clinicians with wide experience of treating crossbite with this type of orthodontic appliance. In order to meet the study objectives, the efficacy of the Invisalign system for expansion movement was determined by statistical analysis, and the predictability of the system’s software (Clincheck) was assessed by comparing planned measurements generated by Clincheck with the real measurements achieved using the transparent aligners by the end of the first treatment phase.

### Inclusion criteria

Inclusion criteria were: patients who had undergone maxillary expansion with SmartTrack aligners; patients of both sexes aged between 18 and 75 years; patients with maxillary compression of non-skeletal, dentoalveolar origin greater than 0.25 mm; patients presenting all permanent teeth (excepting third molars); patients with initial records, as well as final ‘refinement’ or ‘additional aligner’ records obtained with the Itero intraoral scanner; patients who had collaborated adequately in the use of aligners and elastics; patients with or without unilateral or bilateral crossbite; patients treated with intermaxillary elastics without distalization or mesialization of the dental arches; patients who did not undergo corrections in mid-treatment or at later additional aligner phases; patients treated with a minimum of 15 aligners.

### Exclusion criteria

Exclusion criteria were: patients with maxillary compression of more than 15 mm; patients requiring auxiliary expansion appliances; patients with implants, prosthodontic rehabilitations, or ankylosed teeth; patients requiring orthognathic surgery.

### Sample size and groups

Patients were selected from a total of 600 treated patients, applying the inclusion and exclusion criteria detailed above, which left a sample of 114 patients. Patients were classified according to the degree of complexity of the transverse malocclusion, defined by the number of teeth in crossbite and their positions. Special importance was given to cases presenting the second molar in crossbite, as the resolution of these cases is more complex. The sample distribution was as follows: one tooth in crossbite (16.7%), two teeth in crossbite (10.5%), three or more teeth in crossbite (8.8%), bilateral crossbite (9.6%), no crossbite (44.7%), second molar in crossbite (9.6%). Diagnostic records were obtained using the Itero intraoral scanner, both at the start of treatment and at the end of the first treatment phase.

**Fig 1:**
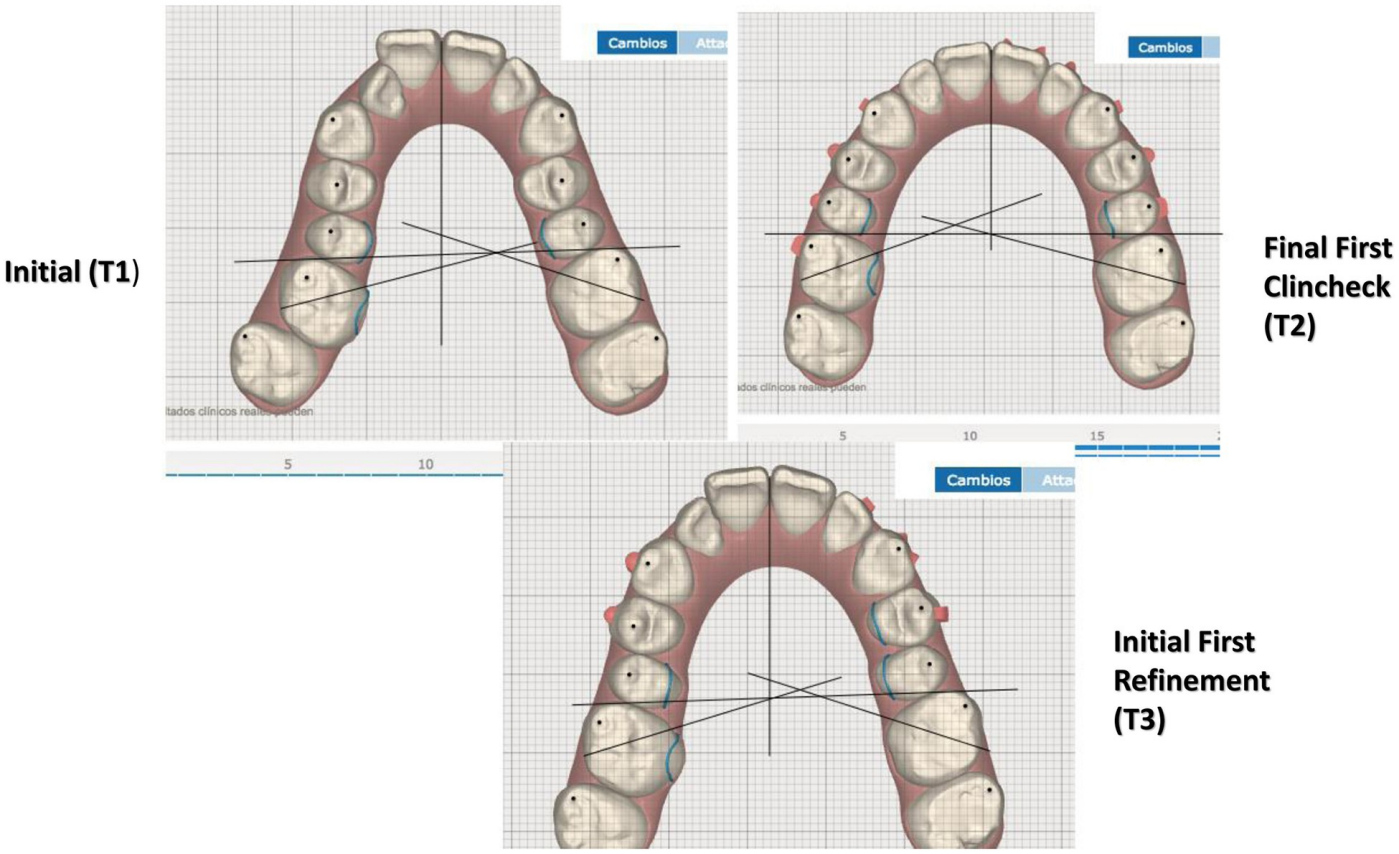
Measurements (canine width, first and second premolar width, first and second molar width, first molar rotation, and first molar inclination) taken at T1 (initial); T2, measurements predicted by Clincheck at end of first treatment phase; T3 measurements at start of first refinement stage.

Clincheck registers were analyzed at three stages (Fig 1): initial dimensions recorded at the start of the first treatment phase (T1); dimensions generated by Clincheck software as predicted measurements at end of first treatment phase (T2); the real dimensions obtained the start of the second treatment phase (also known as “first additional aligners”) (T3).

**Fig 2:**
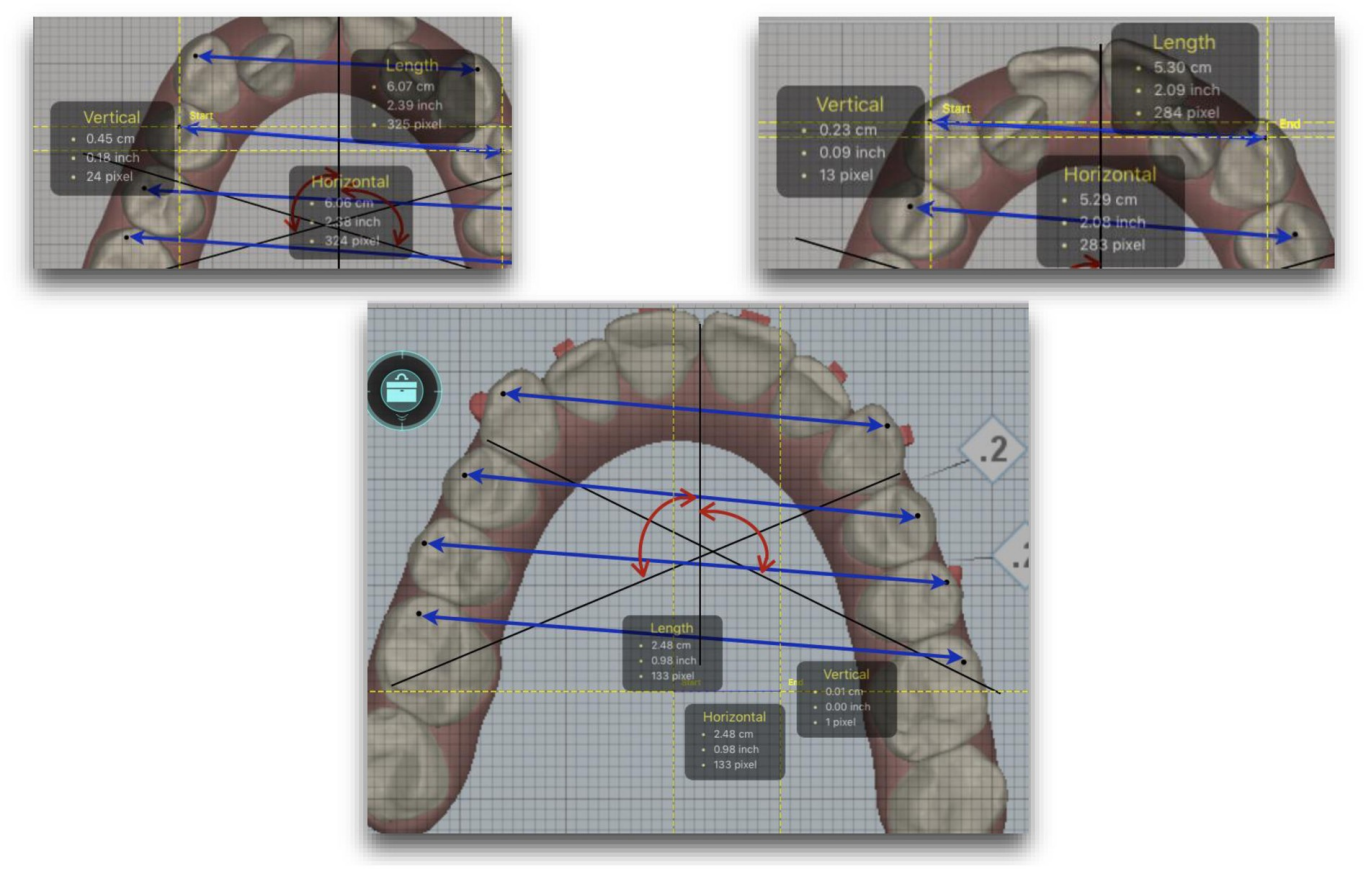
Occlusal images of the maxilla applying the software’s “Grid” tool.

Occlusal images of the maxilla at three stages (T1, T2 and T3) were selected applying the software’s “Grid” tool, which calibrates images by squaring them in millimeters (Fig 2).

**Fig 3:**
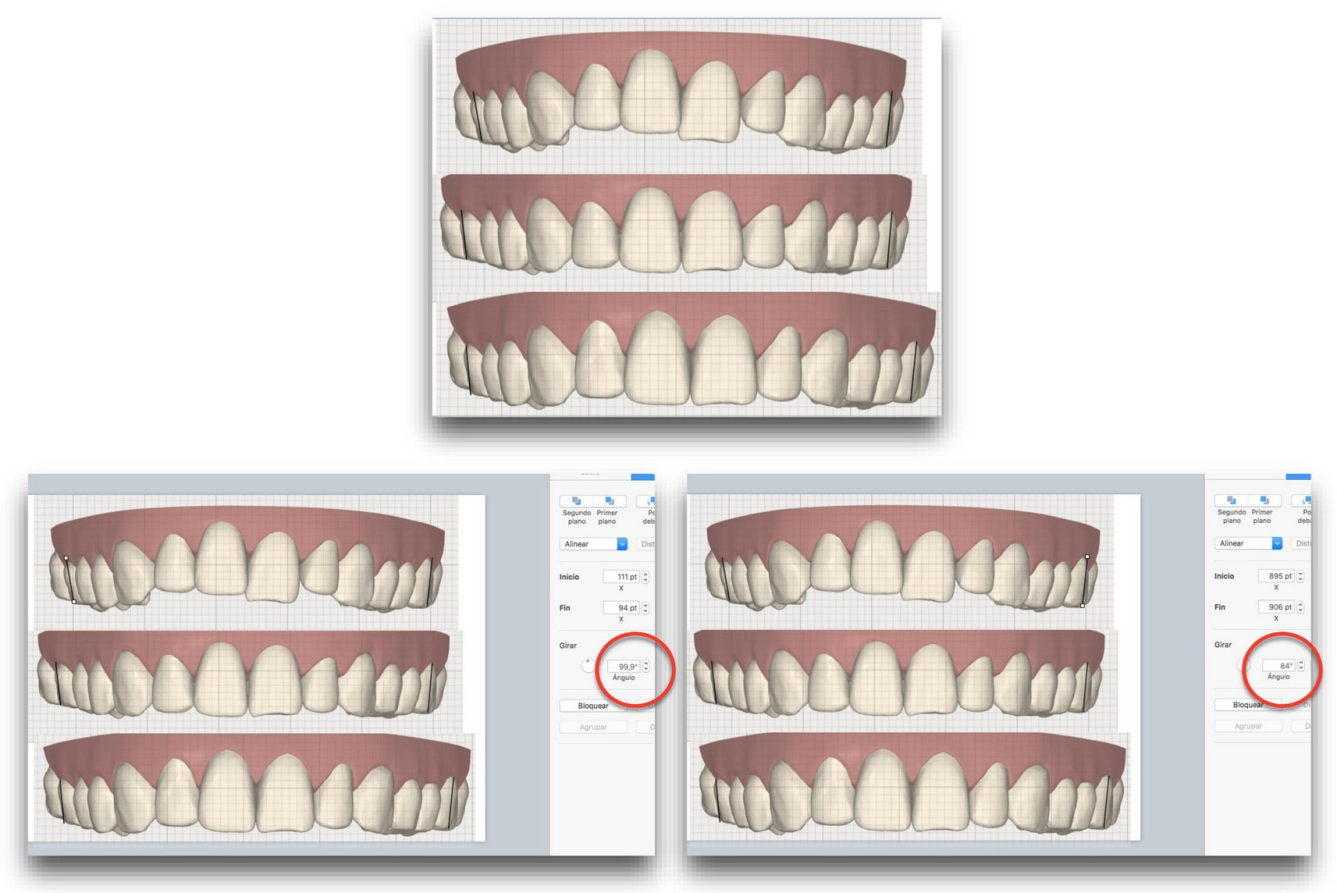
Images exported to Keynote software designed to measure angulation.

These images were exported to a program (Keynote) designed to measure angulation (Fig 3). A series of points are superimposed on each image: for measuring *widths* they are positioned on the vestibular cusps of canines, first and second premolars, and mesio-vestibular cusps of first and second molars; for measuring the *rotation* of the first molar, the angle formed by tracing a line from the disto-vestibular to mesio-palatal cusp of the first molars to the perpendicular line that passes through the point of contact of the central incisors and the horizontal line traced from the tip of the mesio-vestibular cusp of both first molars; for measuring molar *inclination*, the tangent to the vestibular faces of the first molars.

#### Measurements

A computer screen measuring tool RulerSwift for Mac OSX (Apple, Santa Clara, CA, USA) was used to measure angles and distances. This tool measures the distance between two points in inches, millimeters, or pixels, as well as angles in any on-screen image. Measurements were taken from occlusal images captured at T1, T2, and T3.

#### Calibration

RulerSwift was used to calibrate images at T1, T2 and T3 using the Clincheck grid tool.

#### Statistical analysis

The repeated measures t-test was applied to analyze treatment efficacy (differences between means at T3 and T1) and predictability (differences between means at T2 and T3). The normality of the measurements was assessed using the Kolmogorov-Smirnov test. A one-way ANOVA model for repeated measures was estimated to determine whether differences between T3-T1 and T2-T3 depended on the type of malocclusion each patient presented. Multiple comparisons were made with the Bonferroni test.

To study method error or the degree of intra- and inter-observer reproducibility, 25 patients were selected randomly, and the cases were measured by the lead researcher and again by a second examiner. Different indicators of bias and random error were calculated: mean difference, t-test, Dahlberg’s statistic, intra-class variation coefficient, and intra-class correlation coefficient. The significance level applied in analysis was 5% (α = 0.05). Any p-value< 0.05 was indicative of a statistically significant relation; a p-value> 0.05 indicated the absence of a significant relation. Statistical analysis was performed with SPSS v. 24 software (IBM, Armonk, NY, USA).

## Results

The results obtained a very high degree of intra-observer reproducibility (t test p>0.05, Dahlberg values <1 in most of the measurements, coefficient of variation (CV) <2.5%, Intraclass Coefficient Correlation (ICC) >0.9), and inter-examiner reproducibility (t test p>0.05, Dahlberg values <1.5 in most of the measurements, coefficient of variation (CV) <2.5%, Intraclass Coefficient Correlation (ICC) >0.85).

### Analysis of efficacy

Differences between T3 and T1 indicated changes resulting from treatment in the first sequence of transparent aligners (Table 1).

**Table 1:** Linear and angular dimensions at T1 and T3: Mean ± standard deviation, absolute T3-T1 difference and 95% confidence interval, repeated measures t-test, relative difference.

		Mean ± s.d.	T3-T1 (mm)	CI 95%	p-value	T3-T1 (%)
**CANINE**	**T1: PRE**	31.38 ± 2.61	1.87 ± 1.78	1.54–2.20	**<0.001**	+6.31
	**T3: POST**	33.25 ± 2.03				
**1st PREMOLAR**	**T1: PRE**	37.48 ± 3.04	3.14 ± 2.25	2.72–3.56	**<0.001**	+8.73
	**T3: POST**	40.62 ± 2.46				
**2nd PREMOLAR**	**T1: PRE**	42.44 ± 3.49	3.45 ± 2.09	3.06–3.83	**<0.001**	+8.42
	**T3: POST**	45.89 ± 2.84				
**1st MOLAR**	**T1: PRE**	47.12 ± 3.64	2.57 ± 1.83	2.22–2.90	**<0.001**	+5.64
	**T3: POST**	49.69 ± 3.08				
**2nd MOLAR**	**T1: PRE**	55.40 ± 3.84	0.45 ± 1.83	0.11–0.79	**0.010**	+0.54
	**T3: POST**	55.85 ± 3.77				
**INCLINATION right first molar**	**T1: PRE**	-7.77 ± 8.49	2.26 ± 4.76	1.37–3.14	**<0.001**	---
	**T3: POST**	-5.51 ± 8.08				
**INCLINATION left first molar**	**T1: PRE**	-6.54 ± 8.26	2.13 ± 4.09	1.37–2.89	**<0.001**	---
	**T3: POST**	-4.41 ± 7.62				
**ROTATION right first molar**	**T1: PRE**	104.8 ± 4.43	2.22 ± 4.37	1.41–3.03	**<0.001**	+1.87
	**T3: POST**	107.0 ± 3.85				
**ROTATION left first molar**	**T1: PRE**	107.7 ± 4.62	2.46 ± 3.75	1.83–3.30	**<0.001**	+1.95
	**T3: POST**	110.2 ± 4.32				

As the results show, widths underwent significant advances as a result of treatment. Maximum expansion was obtained at the pre-molar level, exceeding 8%. Width at the second molar underwent a smaller expansion (+0.54%). As for *inclinations* and *rotations*, the increase in inclination was significant in every case, and variations in rotation were of around 2%.

Differences between T3 and T1 analyzed by subgroups (no crossbite group and crossbite group) are shown in Table 2. The only significant differences between the two groups were in inclination of the right and left first molars, and rotation of left first molar. For all other variables expansion obtained the same efficacy regardless of group.

**Table 2:** Linear and angular dimensions at T1 and T3 by group (no crossbite group and crossbite group): Mean ± standard deviation, absolute T3-T1 d, repeated measures t-test.

	No Crossbite Group			Crossbite Group			p-value
		Mean ± s.d.	T3-T1 (mm)		Mean ± s.d.	T3-T1 (mm)	
**CANINE**	**T1: PRE**	31.83 ± 2.30	1.54±1.52	**T1: PRE**	31.02 ± 2.81	2.13 ± 1.93	0.076
	**T3: POST**	33.37 ± 1.99		**T3: POST**	33.15 ±2.08		
**1st PREMOLAR**	**T1: PRE**	38.85 ± 2.67	2.82 ± 1.67	**T1: PRE**	37.18 ± 3.30	3.40 ± 2.62	0.173
	**T3: POST**	40.67 ± 2.30		**T3: POST**	40.58 ±2.53		
**2nd PREMOLAR**	**T1: PRE**	42.69 ± 2.98	3.12 ± 1.64	**T1: PRE**	42.24 ± 3.87	3.71 ±2.37	0.132
	**T3: POST**	45.81 ± 2.64		**T3: POST**	45.95 ± 3.02		
**1st MOLAR**	**T1: PRE**	47.08 ± 3.28	2.29 ± 1.53	**T1: PRE**	47.15 ± 3.93	2.79 ± 2.02	0.154
	**T3: POST**	49.37 ± 2.89		**T3: POST**	49.94 ± 3.22		
**2nd MOLAR**	**T1: PRE**	55.28 ± 3.57	0.51 ± 1.22	**T1: PRE**	55.51 ± 4.06	0.39 ± 2.22	0.731
	**T3: POST**	55.79 ± 3.53		**T3: POST**	55.90 ± 3.98		
**INCLINATION right first molar**	**T1: PRE**	-5.98 ± 6.57	1.24 ± 3.22	**T1: PRE**	-9.22 ± 9.57	3.08 ± 5.6	0.039
	**T3: POST**	-4.74 ± 6.16		**T3: POST**	-6.14 ±9.36		
**INCLINATION left first molar**	**T1: PRE**	-5.87 ± 7.29	0.85 ± 2.59	**T1: PRE**	-7.09 ± 8.99	3.17 ± 4.76	0.002
	**T3: POST**	-5.01 ± 6.50		**T3: POST**	-3.93 ± 8.44		
**ROTATION right first molar**	**T1: PRE**	107.0 ± 3.23	1.82 ± 4.14	**T1: PRE**	103.06 ±4.50	2.54 ±4.55	0.386
	**T3: POST**	108.82 ± 3.20		**T3: POST**	105.60 ± 3.74		
**ROTATION left first molar**	**T1: PRE**	111.25 ± 3.33	1.73 ± 3.58	**T1: PRE**	104.76 ± 3.29	3.24 ±4.15	0.042
	**T3: POST**	112.98 ± 2.93		**T3: POST**	108.00 ± 3.98		

### Predictability analysis

Differences between T2 and T3 indicated the precision of Clincheck virtual planning (Table 3). For all widths, virtual planning obtained prognoses of greater expansion than those actually achieved: a mean of 0.63 mm more expansion at the canine level, 0.77 mm at first premolar, 0.81 at second premolar, 0.69 mm at first molar, and 0.25 mm at second molar. All the plan’s estimations, with the exception of the second molar (p = 0.183), were significantly higher than the actual outcomes. As percentages, predictability was 74.8% at the canine level, 80.3% at first premolar, 81% at second premolar, 79.1% at first molar, and 65.2% at second molar.

**Table 3:** Linear and angular dimensions at T2 and T3, mean ± standard deviation, T2-T3 absolute difference and 95% confidence interval, repeated measures t test, relative difference, predictability of the change (%).

		Medan ± s.d.	T2-T3 (mm)	CI 95%	p-value	T2-T3 (%)	Predictability ^a^ (%) mean (T3-T1) / mean (T2-T1)	Predictability ^b^ (%) median
								(T3-T1) / (T2-T1)
**CANINE**	**T2: PLAN**	33.88 ± 2.06	0.63 ± 0.75	0.49–0.77	**<0.001**	+1.93%	74.8%	79.1%
	**T3: POST**	33.25 ± 2.03						
**1st PREMOLAR**	**T2: PLAN**	41.39 ± 2.46	0.77 ± 1.44	0.50–1.04	**<0.001**	+1.96%	80.3%	79.9%
	**T3: POST**	40.62 ± 2.46						
**2**^**nd**^ **PREMOLAR**	**T2: PLAN**	46.70 ± 2.81	0.81 ± 1.26	0.58–1.04	**<0.001**	+1.82%	81.0%	80.9%
	**T3: POST**	45.89 ± 2.84						
**1st MOLAR**	**T2: PLAN**	50.38 ± 3.02	0.69 ± 1.21	0.46–0.91	**<0.001**	+1.43%	79.1%	79.9%
	**T3: POST**	49.69 ± 3.08						
**2nd MOLAR**	**T2: PLAN**	56.10 ± 3.56	0.25 ± 1.97	-0.12–0.61	0.183	+0.54%	65.2%	71.9%
	**T3: POST**	55.85 ± 3.77						
**INCLINATION right first molar**	**T2: PLAN**	-5.94 ± 7.75	-0.42 ± 3.36	-1.05–0.20	0.181	---	123.5%	88.2%
	**T3: POST**	-5.51 ± 8.08						
**INCLINATION left first molar**	**T2: PLAN**	-5.29 ± 7.29	-0.88 ± 2.73	-1.39 –-0.37	**0.001**	---	170.4%	100%
	**T3: POST**	-4.41 ± 7.62						
**ROTATION right first molar**	**T2: PLAN**	107.6 ± 3.86	0.54 ± 3.05	-0.02–1.10	0.059	+0.90%	80.4%	80%
	**T3: POST**	107.0 ± 3.85						
**ROTATION left first molar**	**T2: PLAN**	109.9 ± 4.59	-0.34 ± 3.57	-1.00–0.32	0.309	+0.00%	115.3%	80%
	**T3: POST**	110.2 ± 4.32						

Regarding inclinations, virtual planning was less optimistic for first and second molars, obtaining higher values at T3 than those planned virtually, with significant difference for the upper left first molar. For rotations, planning overestimated the value obtained at the upper right first molar (with a difference close to statistical significance), which corresponded to the real outcome for the upper left first molar.

Differences between T2 and T1 analyzed by subgroups (no crossbite group and crossbite group) are shown in Table 4. No significant differences were observed between the two groups.

**Table 4:** Linear and angular dimensions at T2 and T3 by group (no crossbite group and crossbite group): Mean ± standard deviation, absolute T3-T1 d, repeated measures t-test.

	No Crossbite Group			Crossbite Group			p-value
		Mean ± s.d.	T2-T3 (mm)		Mean ± s.d.	T2-T3 (mm)	
**CANINE**	**T2: PLAN**	33.97 ± 2.05	0.60 ± 0.68	**T2: PLAN**	33.01 ± 2.07	0.66 ± 0.81	0.675
	**T3: POST**	33.37 ± 1.99		**T3: POST**	33.15 ± 2.08		
**1st PREMOLAR**	**T2: PLAN**	41.27 ± 2.26	0.59 ± 1.01	**T2: PLAN**	41.49 ± 2.63	0.91 ± 1.71	0.247
	**T3: POST**	40.67 ± 2.30		**T3: POST**	40.58 ±2.53		
**2nd PREMOLAR**	**T2: PLAN**	46.49 ± 2.64	0.68 ± 1.04	**T2: PLAN**	46.87 ± 2.94	0.92 ± 1.41	0.316
	**T3: POST**	45.81 ± 2.64		**T3: POST**	45.95 ± 3.02		
**1st MOLAR**	**T2: PLAN**	50.06 ± 2.71	0.69 ± 1.07	**T2: PLAN**	50.63 ±3.24	0.69 ± 1.33	0.984
	**T3: POST**	49.37 ± 2.89		**T3: POST**	49.94 ± 3.22		
**2nd MOLAR**	**T2: PLAN**	55.23 ± 3.33	0.25 ± 1.97	**T2: PLAN**	56.24 ± 3.76	0.14 ± 1.12	0.604
	**T3: POST**	55.79 ± 3.53		**T3: POST**	55.90 ± 3.98		
**INCLINATION right first molar**	**T2: PLAN**	-5.73 ± 5.94	-0.42 ± 3.36	**T2: PLAN**	-6.10 ± 9.00	-0.99 ± 2.16	0.104
	**T3: POST**	-4.74 ± 6.16		**T3: POST**	-6.14 ± 9.36		
**INCLINATION left first molar**	**T2: PLAN**	-5.66 ± 6.63	-0.88 ± 2.73	**T2: PLAN**	-5.00 ± 7.83	-0.65 ± 1.72	0.414
	**T3: POST**	-5.01 ± 6.50		**T3: POST**	-3.93 ± 8.44		
**ROTATION right first molar**	**T2: PLAN**	109.14 ± 2.80	0.54 ± 3.05	**T2: PLAN**	106.33 ± 4.16	0.31 ± 2.91	0.470
	**T3: POST**	108.82 ± 3.20		**T3: POST**	105.60 ± 3.74		
**ROTATION left first molar**	**T2: PLAN**	113.10 ± 2.59	-0.34 ± 3.57	**T2: PLAN**	107.29 ± 4.19	0.12 ± 2.73	0.470
	**T3: POST**	112.98 ± 2.93		**T3: POST**	108.00 ± 3.98		

## Discussion

This retrospective study’s null hypothesis was that aligners are not an effective tool for producing arch expansion. On the basis of the results obtained, the null hypothesis was rejected.

All patients were treated by two professionals with wide experience in the use of the Invisalign system. To evaluate the system’s efficacy for arch expansion, patients requiring minimum dentoalveolar expansion of 2.5 mm and treated with a minimum of 15 aligners were selected, who were no longer in growth, of either sex, and were without unilateral or bilateral crossbite. All patients presented molar and canine Class I or slight Class II/III malocclusions treated with elastics alone without molar mesialization or distalization, so that molar displacement would not alter width measurements of the first or second molars. To test the predictability of the Clincheck treatment planning software, measurements were taken at the start of treatment (T1, patient’s initial situation) were analyzed in comparison with the amount of expansion predicted by the software at the end of the first phase or last aligner (T2), and the real expansion produced by aligners at the start of the first refinement/additional aligners (T3).

Regarding the first objective, to determine the efficacy of the Invisalign system for arch expansion, the data obtained indicate that Invisalign transparent aligners are an effective tool for achieving transverse expansion as the results obtained showed an increase in all dental widths to greater or lesser extent. These results are similar to those obtained by other authors [6], who reported an expansion range of 2–4 mm, mainly through the vestibular inclination of crowns, overcorrection being necessary to achieve en masse movement. Another study assessed 51 patients, of who 24 presented transverse problems according to the peer assessment rating index (PAR index) [7]; it was found that 79% obtained improvement through expansion. Results obtained in a study of 77 patients, also using the PAR index, found that 30% of patients presented improvement in comparison with their initial situation [19]. But unlike the present findings and the works cited above, another study found that 71% of 31 patients treated with aligners after orthognathic surgery, underwent changes in transverse expansion; both alignment and buccolingual inclination presented statistically significant post-treatment improvement [20].

The study’s second objective was to assess the predictability of measurements planned using Clincheck software at the end of the first treatment phase with transparent aligners. The literature contains few works that have investigated this software’s predictability. Clincheck generated a prognosis of greater expansion than actually realized, with statistically significant differences in expansion at the level of canines, first and second premolars, and first molars. Second molar expansion was as planned, probably because the second molar was already positioned correctly in 90.4% of the patient sample, making the need for expansion insignificant. Regarding first molar inclination, greater expansion than planned by Clincheck was obtained at tooth 26 (with statistically significant differences), a finding that concurs with most other research, which has observed that expansion results more from coronal-vestibular inclination than en masse movement. Regarding rotations, Clincheck overestimated rotation at 16 (close to significant difference), coinciding with the actual change achieved at 26. Given the large size of the sample (with a confidence level of 94.2%), it is important that differences are not only evaluated on the basis of the results (statistically significant or not) but also in terms of clinical relevance, as small differences may be statistically significant but have minor clinical repercussions.

From the predictability data obtained in the present study (74.8% for canines, 80.3% for first premolars, 81% for second premolars, 79.1% for first molars, 65.2% for second molars) it may be deduced that the aligners’ behavior is similar to that of conventional orthodontic archwires, where it is important to consider inter-tooth distance (termed “inter-bracket distance” in the case of archwires) and the aligner’s deflection (the equivalent of archwire resilience and elasticity).

The study that most closely resembles the present study in terms of sample size (n = 109) reached similar conclusions although the authors did not investigate aligners fabricated from the SmartTrack material and all the patients received additional aligners (from one to five phases of additional aligners), and so the system predictability results were not comparable [10]. Nevertheless, the work studied 64 patients (20 of them with at least one tooth in crossbite) in the first treatment phase without additional aligners, although these were fabricated from the EX30 material [13]. The data showed differences in the predictability of canine expansion (88.7% compared with 74.8% obtained in the present study), but for premolars and molars, predictability data were similar (84.7%, 81.7% and 76.6% compared with 80.3%, 81% and 79.1% obtained in the present work).

Only one other previous work has measured second molar expansion as in the present study, but had a small sample size (n = 30) and failed to specify whether the aligner material was SmartTrack or EX30; moreover, the patients were treated by 22 different clinicians (12 orthodontists and 10 masters’ program students); patients presented slight overcrowding (2 ± 2 mm), treated with interproximal reduction (IPR), reducing the need for expansion; the study also discounted differences of ±0.5 mm and ±2°, which were considered clinically irrelevant [12]. Using a mathematical model of digital superimposition, statistically significant differences were found in the vestibular-lingual movement of second premolars, first molars, and second molars. The main difference was found in the inclination of the second molar with an excess 2° coronal-vestibular inclination, due to the reduction in force exerted at the end of the aligner action because of the greater elasticity in this area.

Various studies [10, 11] have quantified expansion at the level of the gingiva, but we consider these data invalid as, during Clincheck’s planning process (digital detailing of impressions, section and individualization of each tooth, the software’s “Treatment” tool and virtual gum positioning), technicians eliminate the gum from the digital model before the software calculates all the parameters and protocols, and random virtual positioning of the gingiva is the last step to be performed without applying any specific criteria, which means that the virtual gingiva will differ from one technician to another. For this reason, gingival data cannot be exact and will vary from one Clincheck outcome to another.

One of the main limitations of the present work resides in the measurement methods used. Rather than the manual measurements taken from 2D images, it would be possible to take measurements from 3D digital models by superimposing STL files. It would also be more precise to evaluate changes three-dimensionally using CBCTs, although this would mean exposing patients to radiation, which can be considered ethically questionable. Another topic for investigation is the influence of the use of elastics on the predictability of molar expansion movement in patients presenting crossbite, where this movement tends to be less effective.

The present study had a large sample of 114 patients treated during the first treatment phase, without additional aligners, assessing the efficacy of the Invisalign system using Smart Track material and the predictability of Clincheck software for expansion movement. As other authors have suggested, it would be useful to apply some overcorrection and investigate the use of intermaxillary elastics, and the presence or absence of attachments for this type of movement, so further research is needed to investigate these parameters.

## Conclusions

- Aligners are an effective tool for producing arch expansion, being more effective in premolar area and less effective in canine and second molar area.
- Predictability was reasonable for expansion movement.
- Based on these results, overcorrection should be considered at the virtual planning stage in order to obtain the expected outcomes.

## Supporting information

S1 Data (XLS)
